# Pathological and Diagnostic Assessment of Duodenal Wound Healing: A Comparative Experimental Study of Jejunal Serosal vs. ePTFE Patch Repair

**DOI:** 10.3390/medicina62010171

**Published:** 2026-01-14

**Authors:** Ilija Golubovic, Milan Radojkovic, Ivan Ilic, Vladimir Petrovic, Marko Stojanovic, Jelena Zivadinovic, Aleksandar Vukadinovic, Nebojsa Ignjatovic

**Affiliations:** 1 Clinic for Digestive Surgery, University Clinical Center Nis, 18000 Nis, Serbia; radojkovici71@gmail.com (M.R.); acavuk89@gmail.com (A.V.); n.ignjat@gmail.com (N.I.); 2 Center for Pathology and Pathological Anatomy, University Clinical Center Nis, 18000 Nis, Serbia; ilicko81@gmail.com; 3 Faculty of Medicine, Institute for Embryology and Histology, 18000 Nis, Serbia; vladimir.petrovic@medfak.ni.ac.rs; 4 Clinic for Gastroenterology and Hepatology, University Clinical Center Nis, 18000 Nis, Serbia; marcss994@gmail.com; 5 Clinic for Anesthesiology and Intensive Therapy, University Clinical Center Nis, 18000 Nis, Serbia; jelena5491@gmail.com

**Keywords:** severe duodenal injuries, surgical treatment, jejunal serosal patch, expanded polytetrafluoroethylene patch, an experimental study, histopathological assessment, wound repair

## Abstract

*Background and Objectives:* The treatment of duodenal injuries remains one of the most challenging issues in clinical surgery due to their high morbidity and mortality rates. The primary objective of this study was to evaluate the histopathology and other diagnostic outcomes of wound repair following surgical reconstruction of large experimental duodenal defects using synthetic (ePTFE, expanded polytetrafluoroethylene) or organic (JSP, jejunal serosal patch) materials. *Materials and Methods:* A total of 20 European rabbits were randomly divided into two equal groups (*n* = 10 each). A grade III defect covering over 50% of the duodenum’s circumference was created in the second part of the duodenum of the rabbits. The anesthesia, duodenal injury, postoperative care, and animal sacrifice protocols were identical for all experimental rabbits. The effectiveness of JSP and ePTFE patch repair techniques was investigated based on clinical, macroscopic, and microscopic assessments at two and four weeks postoperatively. *Results:* Survival rates were comparable between groups (*p* > 0.05). Remarkable mucosal regeneration was evident in all experimental animals by two weeks, showing complete coverage of the jejunal serosal and ePTFE patches by re-epithelialized mucosa with functional villus formation. While partial development of the underlying muscular and serosal layers was observed in both groups at four weeks, the JSP group achieved a significantly higher median histological score (19 vs. 14; *p* = 0.003). Conversely, the ePTFE group exhibited a major safety concern: a highly significant increase (*p* ≤ 0.001) in Grade 4 dense, inseparable adhesions throughout the abdominal cavity, which were entirely absent in the JSP group. *Conclusions:* Both JSP and ePTFE are viable for duodenal reconstruction, but the autologous JSP is superior in tissue healing and safety. Severe adhesions associated with ePTFE constitute a significant clinical concern, limiting its use to a second-line alternative. Consequently, JSP is the preferred option, while ePTFE requires further long-term safety validation.

## 1. Introduction

The management of duodenal injuries is one of the most significant challenges in clinical surgery due to their high reported rates of morbidity (up to 60%) and mortality (up to 25%). These severe outcomes are primarily driven by complications following surgical repair, such as duodenal fistulas and sepsis, which can lead to multiple organ failure [1]. Duodenal wall defects commonly result from penetrating or blunt abdominal trauma. However, they are also increasingly associated with iatrogenic causes, including complications from treatments for colon, duodenum, or right kidney tumors. They are also associated with laparoscopic cholecystectomy and various endoscopic procedures, such as endoscopic retrograde cholangiopancreatography (ERCP) and gastroscopy [2,3,4].

While most minor duodenal injuries can be successfully treated with primary closure of the defect (duodenorrhaphy), large, severe defects classified as Grade III or higher, which cover over 50% of the duodenal circumference, present a significantly greater challenge [5,6]. The primary concern in these severe cases is the high risk of suture line dehiscence, which requires promoting effective healing while preventing life-threatening complications. Due to these risks, considerable research over the past two decades has focused on finding effective alternative surgical techniques for complex duodenal reconstruction [1,7,8,9,10,11].

Over time, the jejunal serosal patch (JSP) technique has become a preferred autologous option for repairing large duodenal defects in experimental models due to its consistent positive outcomes [7,8,12]. Meanwhile, synthetic grafts, such as expanded polytetrafluoroethylene (ePTFE) patches, have emerged as an alternative for restoring full-thickness defects in the alimentary tract [7,11,13,14]. While ePTFE is readily available and easy to handle, its use in duodenal repair is met with clinical skepticism. This has resulted in limited and often conflicting data regarding the comparative efficacy and safety of repairing duodenal wall defects using organic JSP versus synthetic ePTFE material [11,13].

To date, comparative experimental studies evaluating JSP and ePTFE for treating severe duodenal defects have primarily focused on animal survival rates and qualitative, descriptive assessments of the healing process [7,8,10,11]. However, there remains insufficient data for a detailed quantitative analysis of defect healing and tissue regeneration. The primary objective of this study was therefore to quantitatively compare the clinical, macroscopic, and microscopic (histopathological scoring) outcomes of JSP and ePTFE repair methods for reconstructing severe experimental duodenal defects in an animal model.

## 2. Materials and Methods

### 2.1. Experimental Animals

This experimental research was conducted after receiving approval from the Ethical Committee of the Faculty of Medicine at the University of Niš and the Veterinary Ad-ministration of the Ministry of Agriculture and Environmental Protection (approval number 323-07-00073/2017-05/9). Twenty European rabbits (*Oryctolagus cuniculus*), weighing between 2.5 and 3 kg, were obtained from the Department for the Breeding of Laboratory and Experimental Animals at the Military Medical Academy in Belgrade, Serbia. The study was conducted at the Department of Experimental Medicine at the Scientific Research Center for Biomedicine at the Faculty of Medicine, University of Niš in Serbia. The rabbits were placed in individual cages and kept at room temperature. All experimental animals were given unrestricted access to regular food and tap water prior to the experiment. The protocols for anesthesia, duodenal injury, postoperative care, and animal sacrifice were identical for all experimental rabbits. The animals were treated according to the Guide for the Care and Use of Laboratory Animals, published by the National Research Council Committee in 2011 [15].

### 2.2. Anesthesia Protocols

For sedation and analgesia, medetomidine hydrochloride (0.25 mg/kg) was administered intramuscularly, and its effects were observed after 10–15 min. Then, 10–20 min later, we administered an intramuscular anesthetic, ketamine 10% (25 mg/kg), on the opposite side from the previous injection. This allowed for spontaneous breathing. To counteract the sedative effects of medetomidine hydrochloride, atipamezole hydrochloride (0.25 mg/kg) was administered intramuscularly immediately postoperatively, upon completion of the skin closure, while the animal was still on the operating table. The animals returned to a normal state within 5–10 min. Occasionally, it may be necessary to administer atropine subcutaneously (0.05 mg/kg) to prevent bradycardia.

Intraoperative Monitoring and Support: Throughout the surgical procedure, the depth of anesthesia was closely monitored by assessing the palpebral and pedal withdrawal reflexes. Vital signs, including heart rate, respiratory rate, and oxygen saturation, were monitored using a pulse oximeter placed on the ear. To prevent hypothermia, which is critical for successful recovery, the animals’ core body temperature was maintained within the physiological range (37–38.5 °C) using a controlled electric heating pad. Sterile ophthalmic lubricant was applied to prevent corneal drying.

### 2.3. Surgical Techniques and Experimental Groups

The surgical procedures were performed under aseptic conditions after an overnight fast. Up to one hour preoperatively, the animals received an intramuscular injection of enrofloxacin antibiotic (5 mg/kg). After making a superior midline incision, a large duodenal defect was created in the second part of the duodenum by excising a portion of the anti-mesenteric wall that covered over 50% of the total circumference (AAST Grade III). The defect was 20 mm long. The diameter of the duodenal lumen was measured at the site of the defect in all animals. To allow for sufficient contact between the duodenal juice and the wound edges and local visceral peritoneum, we waited 30 min. This controlled contamination period was maintained to create a model of severe, delayed duodenal injury. The rabbits remained under continuous general anesthesia and the abdominal cavity was covered with sterile, moist surgical drapes throughout the entire 30 min interval.

There were ten rabbits in each group. For group 1, a loop of proximal jejunum within the first 30 cm of the Treitz ligament was used as a JSP to cover the duodenal defect. This technique involved circumferential seromuscular suturing through the uninjured part of the duodenal wall. Group 2 underwent repair of the duodenal injury using an ePTFE patch (Gore-Tex; W. L. Gore & Associates, Flagstaff, AZ, USA), which was sewn circumferentially through the uninjured part of the duodenal wall to cover the duodenal defects. Both techniques used a 6-0 glyconate suture (Monosyn^®^, B. Braun Surgical, S.A., Barcelona, Spain).

The superior midline incision was closed with a continuous 3-0 polypropylene suture for the fascia. The skin and attached subcutaneous tissue were sutured with a 2-0 polypropylene suture (Prolene^®^, Ethicon, Edinburgh, Scotland) and a 3-0 glyconate suture (Monosyn^®^, B. Braun Surgical, S.A., Barcelona, Spain), respectively.

### 2.4. Postoperative Care and Monitoring

After the procedure, all of the rabbits were closely monitored in separate cages. A dedicated veterinary technician conducted clinical monitoring twice daily for signs of pain, distress, or infection (e.g., decreased appetite, lethargy, wound swelling).

The antibiotic enrofloxacin was administered at a dose of 5% (5 mg/kg) by mouth twice daily for seven days. Postoperative pain management was prioritized to ensure animal welfare and optimize recovery. Meloxicam, a non-steroidal anti-inflammatory drug (NSAID), was administered to provide preemptive and sustained analgesia. The dosage used was 0.2–0.6 mg/kg of body weight, administered subcutaneously immediately after the incision was closed. This dosage was repeated every 24 h for a minimum of five postoperative days, or as needed based on clinical assessment of pain.

Water and food were provided ad libitum on the first and second days after surgery, respectively. Other care was according to routine.

The tenth day was used as the criterion for survival. During the postoperative period, the animals’ activity was monitored daily until they were sacrificed, and their body weight was measured. Evidence of poor feeding and obstipation was also recorded.

### 2.5. Gross Pathology

Half of the rabbits from each experimental group were sacrificed at the end of the second and fourth weeks, respectively. The rabbits were euthanized with an intravenous administration of a drug: T-61^®^ (Intervet International GmbH, Unterschleissheim, Germany), at a dose of 0.3 mL/kg (embutramide 200 mg/mL + mebezonium 50 mg/mL + tetracaine 5 mg/mL). After sacrifice, the abdomen was opened and the abdominal cavity and duodenum were examined to record the following parameters: intestinal obstructions, abscesses, and adhesion of various degrees. The luminal diameter of the duodenum was measured again at the healing site. Postoperative adhesions were evaluated and graded according to the classification system reported by Saygun et al. (2006) [10]:

- Grade 1: Scattered, filmy adhesions.
- Grade 2: Moderately dense, scattered adhesions that are easily separated.
- Grade 3: Dense, continuous adhesions that are easily separated.
- Grade 4: Very dense, homogeneous adhesions that are not easily separated.

### 2.6. Histopathological Examinations

All tissue specimens were sampled and fixed in 10% formalin. The specimens were then embedded in paraffin and cut into 3-μm-thick serial sections. After mounting on slides and deparaffinizing, the specimens were stained with hematoxylin and eosin. A pathologist who had no information about the harvesting period of the rabbits performed all histopathological examinations. The histopathological findings were the result of analyzing each sample from rabbits sacrificed in the second and fourth weeks. Wound healing was assessed using the modified histological scoring system reported by Ziaian et al. (2014) [8]. Six histological parameters were evaluated on a scale of 0 to 4, where higher scores generally indicate better healing outcomes:

- Epithelialization: Scored as 0–1 (none), 2 (partial), 3 (complete but immature), or 4 (complete and mature).
- Collagenization: Scored as 0–1 (none), 2 (partial), 3 (complete but irregular), or 4 (complete and regular).
- Inflammation: Graded inversely as 0 (severe), 1 (moderate), 2 (mild), or 3–4 (none).
- Neovascularization: Assessed by the number of vessels per high-power field (HPF) and scored as follows: 0–1 (none); 2 (<5/HPF); 3 (6–10/HPF); or 4 (>10/HPF).
- Necrosis: Graded as 0 (extensive), 1 (focal), or 2–4 (none).
- Granulation tissue: Scored as 0 (none), 1 (immature), 2 (mildly mature), 3 (moderately mature), or 4 (fully mature).

### 2.7. Statistical Analysis

Statistical significance was analyzed using a suite of comparative tests. The choice of test—Student’s *t*-test or Mann–Whitney U test—depended on the type of observations and data distribution. The Shapiro–Wilk test was used to evaluate the normality assumption of the data distribution. Data are expressed as mean ± standard deviation (SD), with the exception of frequencies and categorical variables (e.g., adhesion grades). The key metric for tissue regeneration was the total score, which is the sum of the numerical scores obtained from six defined histological parameters using the modified histological scoring system. Statistical significance was defined as a *p*-value of *p* ≤ 0.05, *p* ≤ 0.01, or *p* ≤ 0.001.

Data analyses were performed using the statistical software packages IBM^®^ SPSS^®^ Statistics 27.0 (IBM Corp., Armonk, NY, USA) and Microsoft Excel 365.3 (Microsoft Corp., Redmond, WA, USA).

## 3. Results

### 3.1. Study on Survival, Animal Activity, Evidence of Poor Feeding, Obstipation and Body Weight

All of the animals survived the experiment. No significant change in activity was observed in any animal. Full feeding was observed in all animals on the second postoperative day. No evidence of obstipation was found during the experiment. The increase in body weight of the rabbits was significant over time. Table 1 shows the summary statistics and statistical significance of the animals’ body weight before surgery and at the end of postoperative weeks two and four (*p* ≤ 0.05 and *p* ≤ 0.001, respectively).

### 3.2. Gross Pathology (Intra-Abdominal Abscess, Obstruction, Duodenal Lumen, Adhesions)

After opening the abdomen of each sacrificed animal, none showed an intra-abdominal abscess or intestinal obstruction. There were no significant decreases in the luminal diameter of the duodenum after surgery in any rabbit (Table 1).

**Figure 1:**
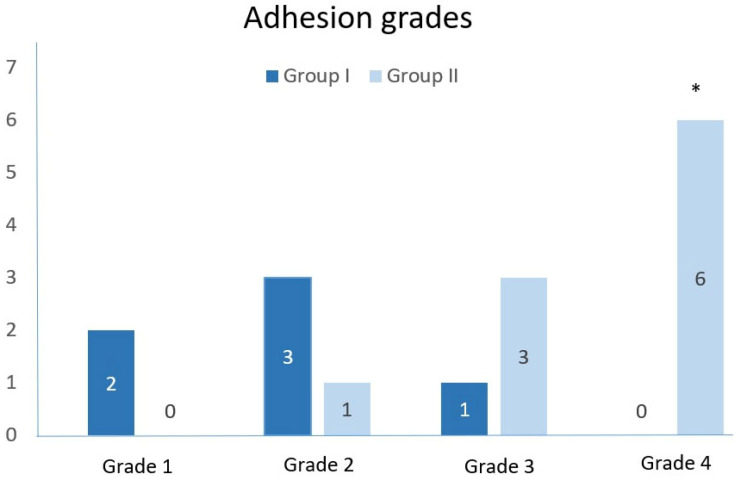
Final distribution of intra-abdominal adhesion grades. Data represent the findings recorded at the time of sacrifice, specifically at the 2-week and 4-week post-surgery intervals. * The distribution of adhesion grades was significantly different between the two groups (Mann–Whitney U test, *p* ≤ 0.001). Adhesion grades of the experimental animals: Grade 1: scattered, filmy adhesions; Grade 2: moderately dense, scattered adhesions that are easily separated; Grade 3: dense continuous adhesions that are easily separated; Grade 4: very dense homogeneous adhesions that are not easily separated.

Group I (JSP) showed dominant partial adhesions to the proximal jejunum, with no adhesions found in four (40%) of the experimental animals. In contrast, partial adhesions to the liver were detected in seven (70%) of the rabbits in group II (ePTFE patch). Figure 1 illustrates the differences in adhesion grades among the experimental animals. Statistical analysis using the Mann–Whitney U test showed a significant difference in the distribution of adhesion grades between the two groups (*p* ≤ 0.001). Specifically, the ePTFE group exhibited a trend toward more severe adhesions, with 6 cases of Grade 4 adhesions (very dense, homogeneous, and difficult to separate), which were notably absent in the JSP group.

### 3.3. Micro Pathology (Images, Score System)

A gross examination at four weeks revealed that the jejunal serosal patches remained in place. All rabbits had intact ePTFE patches, with the margins inverted into the lumen. In both groups, the margin of the duodenal wall defect was thicker due to mild inflammation.

### 3.4. The Results of the JSP Group

**Figure 2:**
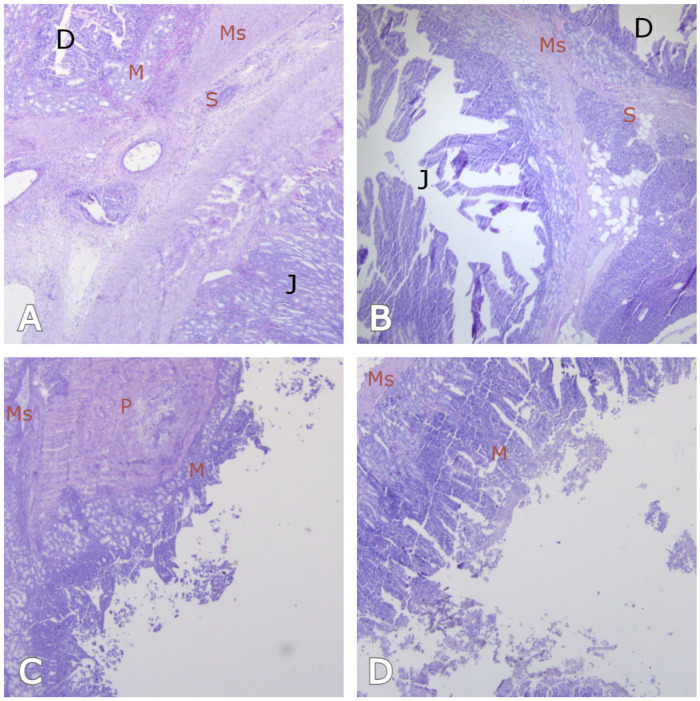
Mucosal (M) regeneration, but no muscular (Ms) or serosal (S) regeneration was observed in all animals at the end of the 2nd week (**A**,**C**). At the end of the 4th week, complete coverage of duodenal re-epithelialized mucosa (M) on the jejunal surfaces and ePTFE patches was observed. Partially developed muscular and serosa were observed in all animals of both groups at the same period (**B**,**D**). (Hematoxylin-eosin stain, original magnification ×40). Abbreviations and Labels: D, *Duodenum*; J, *Jejunum*, P, *Patch of expanded polytetrafluoroethylene with graft tissue*.

By the end of the second week, all of the animals in the JSP group had shown mucosal regeneration, though there was no muscular or serosal regeneration (Figure 2A). Re-epithelialized mucosa ingrowth began along the margins of the serosal surface of the jejunal patch. By four weeks, complete duodenal re-epithelialized mucosa coverage of the jejunal surfaces was observed. At the same period, partially developed muscle and serosa were observed in all animals of both groups (Figure 2B). At the two-week interval, the inflammatory infiltrate in the JSP group was predominantly acute, characterized by the presence of neutrophils and focal edema around the wound edges. By the fourth week, this shifted to a mild chronic inflammatory pattern, consisting mainly of lymphocytes and plasma cells, coinciding with advanced tissue repair.

### 3.5. The Results of the ePTFE Group

Histological studies confirmed the gross observations. Over time, a layer of mesothelial cells progressively covered the outer layer of the ePTFE patches. At 2 weeks, columnar epithelial cells with typical regenerative patterns, granulation tissue with new angiogenesis, immature mesenchymal tissue, and smooth muscle proliferation were observed. However, the re-epithelialized mucosa exhibited rudimentary villous structures and a lack of mature goblet cells (Figure 2C). At four weeks after surgery, complete coverage of the ePTFE grafts by re-epithelialized mucosal surface consisting of columnar epithelium with villus formation was observed in all experimental animals. Furthermore, the serosa, scar, and fibrous tissue covered the defect site, as did the muscular layer. Histological evaluation revealed nearly complete smooth muscle formation and a mild chronic inflammatory reaction (Figure 2D). Similarly to the JSP group, the ePTFE group showed an initial acute inflammatory response at two weeks. However, at four weeks, a mild but persistent chronic inflammatory reaction was observed around the synthetic fibers, characterized by lymphocytes and occasional foreign-body giant cells. Despite the presence of the synthetic material, the overall qualitative nature of the inflammation did not differ significantly from the autologous JSP repair, and it did not clinically impede the healing process.

### 3.6. Comparative Analysis

**Figure 3:**
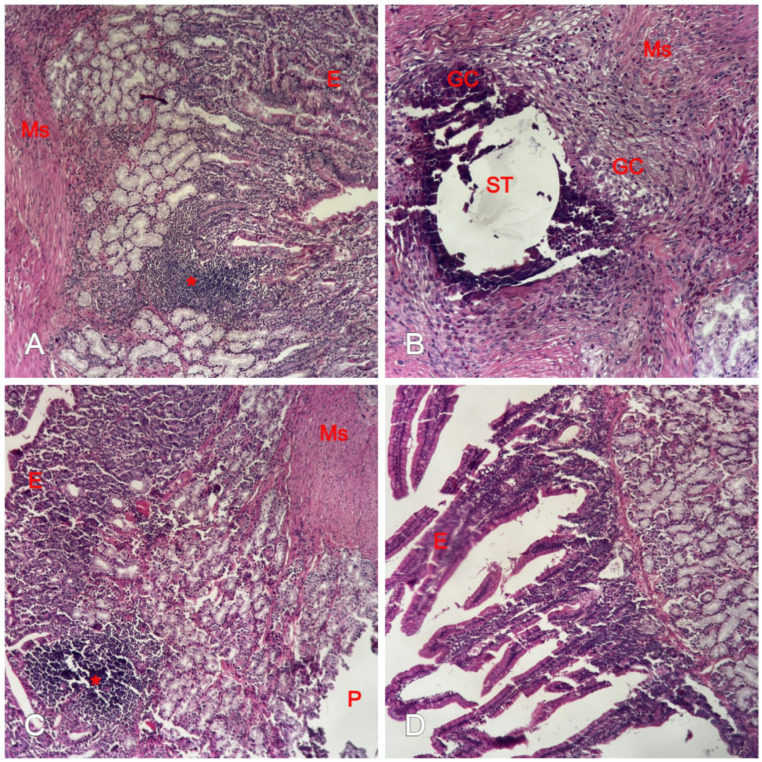
(**A**) JSP group: Complete and mature epithelialization (E) with well-developed functional villi and crypts. The repair site exhibits an organized inflammatory infiltrate (*) composed primarily of lymphocytes, indicating a resolved inflammatory phase and excellent tissue integration. (**B**) JSP group: Advanced regeneration of the muscular layer (Ms); newly formed smooth muscle fibers demonstrate orderly linear orientation and structural continuity. Visible suture tracks (ST) are present, characterized by small tissue voids surrounded by occasional foreign-body giant cells (GC), representing a localized and isolated reaction to the suture material itself. (**C**,**D**) ePTFE group: Mature columnar epithelium (E) bridging the synthetic graft area. The underlying layers show a persistent, mild chronic inflammatory response (reflecting a lower inflammation score) and disorganized fibromuscular tissue compared to the JSP group. A mucosal defect/void is visible, which occurred as a processing artifact due to the detachment of the ePTFE graft (P) during histological preparation. (Hematoxylin-eosin stain; original magnification ×100 for **A**,**C**; ×200 for **B**,**D**). Abbreviations and Labels: P—*patch site/void (originally occupied by ePTFE)*; ST—*suture track*; E—*epithelium*; Ms—*muscularis*; GC—*foreign-body giant cells*; *—*organized lymphocytic infiltrate*.

In both experimental groups, the regenerated re-epithelialized mucosa by the fourth week reached full epithelial maturation, characterized by a well-organized villus architecture with functional crypts. Histological analysis confirmed the presence of goblet cells within the columnar epithelium, indicating that the re-epithelialized mucosa had acquired its necessary secretory and absorptive functions. Regarding the regeneration of the muscular layer, a similar pattern was observed in both the JSP and ePTFE groups by the fourth week. The initial regenerative phase at two weeks was characterized by a predominately disorganized fibromuscular tissue, where myofibroblasts and collagen fibers were interspersed with immature muscle cells. However, by the end of the study (four weeks), there was clear evidence of more orderly regeneration. The newly formed smooth muscle fibers began to demonstrate a more linear orientation and continuity, although they did not yet reach the thickness or perfect organization of the native *tunica muscularis*. This suggests a progressive transition from early scarring to organized myogenic repair in both repair techniques. The superior regenerative capacity of the JSP technique, characterized by more mature tissue architecture and organized inflammatory response, is further illustrated in the high-magnification images in Figure 3.

The modified wound healing histological scoring system was applied to the histological examinations. The median total score for the JSP group was 11 at two weeks and 19 at four weeks. These scores were higher than those of the ePTFE group (9 at two weeks and 14 at four weeks). Statistical analysis revealed a highly significant difference in the median total histological scores between the JSP and ePTFE groups (*p* = 0.003, Mann–Whitney U test). These findings, detailed in Table 2, demonstrate the superior regenerative capacity of the JSP technique.

## 4. Discussion

Morbidity and mortality rates following duodenal injury primarily depend on duodenal fistulas resulting from suture dehiscence, which can lead to sepsis and multiple organ failure [1,16]. Our experimental model demonstrated the short-term clinical safety of both repair methods. Specifically, there was no mortality, anastomotic dehiscence, or other major complications observed in any of the experimental animals. These highly favorable results align with those reported in previous experimental studies evaluating these patch repairs [7,8,10,11]. Furthermore, both test groups showed comparable and significant weight gain over the postoperative period, with no evidence of poor feeding or obstipation, confirming the functional success of the reconstructions.

Simple primary closure (duodenorrhaphy) is sufficient for most minor duodenal injuries. However, alternative or additional surgical techniques are necessary for man-aging large, severe defects (Grade III or higher), as there is a substantial risk of suture line dehiscence, excessive tension, and duodenal luminal narrowing [2,5,6,17,18]. Reconstructive options include jejunal serosal patching (JSP), Roux-en-Y duodenojejunostomy, pedicled grafts, duodenal resection with end-to-end duodenoduodenostomy, diverticulization, pyloric exclusion, and the Whipple procedure [5,7,8,11,19,20,21]. This study investigated the comparative effectiveness of two promising techniques for reconstructing severe experimental duodenal defects: the autologous JSP and the synthetic ePTFE patch.

Since Kobolt and Thal [22] reported the JSP method for managing experimental duodenal wounds in dogs in 1963, it has yielded encouraging outcomes [7,8,10,11,12,22]. The JSP can be performed by suturing a bowel loop over the defect (e.g., an Omega loop) [23] or as a Roux-en-Y jejunal loop. However, the Roux-en-Y jejunal loop has been criticized due to the formation of a new suture line [24]. The main advantage of the JSP method is its use of an autologous graft, which does not increase the risk of infection compared to an ePTFE patch [25,26,27,28].

A primary finding that distinguished our study was the significant difference in adhesion formation between the two groups. Specifically, the ePTFE group showed a statistically significant increase in Grade 4 adhesions (very dense and homogeneous, which are difficult to separate) compared to the JSP group (*p* ≤ 0.001). In contrast, the JSP group predominantly showed less severe, partial adhesions. This observation highlights a major drawback of the synthetic ePTFE material in the abdominal environment. Although ePTFE is designed to be inert, its persistent presence as a foreign body, coupled with its surface characteristics, may trigger an aggressive, extensive inflammatory and fibrotic response, contributing to the observed dense adhesions. These findings are highly relevant clinically, as severe adhesions can lead to complications such as chronic abdominal pain and small bowel obstruction, suggesting a potential long-term risk associated with the ePTFE patch [11,14,29,30,31].

The primary scientific contribution of our study lies in its quantitative comparison of histological scores of wound healing between autologous (JSP) and synthetic (ePTFE) materials. This comparison provides a precise metric for evaluating the regenerative process. The quantitative histological analysis revealed a clear advantage of the autologous JSP over the synthetic ePTFE patch. While both materials promoted mucosal regeneration, the JSP group achieved a significantly higher median total histological score by the fourth week (19 vs. 14, *p* = 0.003). This highly significant difference suggests that the biological properties of the jejunal serosa provide a more inductive environment for organized tissue repair compared to the synthetic ePTFE scaffold. Complete re-epithelialized mucosal coverage and functional villus and crypt formation were observed by the four-week endpoint in all animals. This rapid epithelialization aligns with previous pathohistological reports [7,8,10,11,14]. However, the regenerative process was incomplete for the deeper layers. No muscular or serosal regeneration was evident at the two-week endpoint, and only partial development of the muscular and serosal layers was present at the four-week endpoint (Figure 2B,D). The incomplete regeneration of the muscularis propria observed at four weeks may have several clinical implications. While the re-epithelialized mucosa provides an adequate anatomical barrier, the lack of a fully organized and thick muscular layer could potentially lead to the formation of duodenal pseudodiverticula or localized dysmotility in the long term. Furthermore, this structural thinning might represent a *locus minoris resistentiae*, although in our study, no late perforations or mechanical failures were observed. Future long-term studies are required to determine if the muscular layer eventually achieves full thickness and functional strength.

In addition to the satisfactory results of previous studies on replacing a segment of the intestinal wall with JSP [7,8,12,23], it is important to note that a new repair technique using synthetic grafts (ePTFE) has been developed [10,11,13,25,32]. Several studies have tested the efficacy of ePTFE patches for repairing defects in the alimentary tract wall. Oh et al. (2002) demonstrated the temporary successful replacement of full-thickness intra-abdominal hollow viscus defects with ePTFE patches in their experimental study [14]. Previous studies have also presented positive results from the use of ePTFE patches in repairing large duodenal defects [7,10,11]. A comparative study by Nikeghbalian et al. (2008) revealed that the ePTFE patch method is comparable to the JSP method for reconstructing severe experimental duodenal defects [11]. Furthermore, the ePTFE patch can serve as a reliable barrier against duodenal contents, albeit only temporarily. As a result, a unique collagen matrix emerges, facilitating the migration of new cells [33]. We confirmed that the resulting re-epithelialized mucosal surface was functional. This was evidenced by the presence of villi and crypts, as well as the maintenance of normal body weight in the animals. Importantly, neither the JSP nor the ePTFE group showed a significant postoperative reduction in duodenal luminal diameter, indicating that neither technique compromised luminal patency.

The use of ePTFE is appealing because of its specialized biomaterial properties, such as high chemical stability, mechanical strength, biocompatibility, and affordability [28]. The material’s highly organized microporous architecture, defined by a pore size of approximately 30 μm (the internodal distance), serves two functions. First, it promotes post-implantation tissue integration through fibrocollagenous ingrowth. Second, it effectively impedes the migration of secretions [32]. However, the study’s short follow-up period of four weeks is a limitation because the long-term integrity and functional outcome of the partially regenerated muscular and serosal layers remain un-known. While ePTFE demonstrates functional efficacy in terms of mucosal coverage, the high incidence of Grade 4 adhesions (*p* ≤ 0.001) raises significant safety concerns. These dense, inseparable adhesions are not merely a disadvantage but a potential precursor to severe clinical complications, such as chronic pain, small bowel obstruction, or extreme difficulty in re-operative surgery. Consequently, the clinical translation of ePTFE for duodenal repair must be approached with extreme caution, as the biological benefits of mucosal healing may be outweighed by these substantial macroscopic risks. Finally, a significant limitation is the inherent difficulty in extrapolating these preclinical findings from a rabbit model to human clinical scenarios. While rabbits are widely used in experimental surgery, their smaller anatomical scale and different gastrointestinal physiology compared to humans necessitate caution before these patch repair techniques are widely applied in clinical practice. Therefore, additional experimental and preclinical trials with longer follow-up periods are necessary to confirm the long-term clinical utility of ePTFE patches for repairing large duodenal defects and to determine the definitive standard clinical application.

## 5. Conclusions

In summary, while both JSP and ePTFE techniques are technically feasible for the reconstruction of severe duodenal defects, our study demonstrates that the JSP is superior in terms of histological wound healing and reduced complication profiles. Statistical analysis confirmed that JSP achieves significantly higher regenerative scores by the fourth week (*p* = 0.003). Furthermore, the JSP group showed a markedly lower risk of dense intra-abdominal adhesions compared to the ePTFE group. Therefore, although ePTFE remains a functional alternative, the autologous JSP should be considered the preferred surgical option for complex duodenal repair due to its superior tissue integration and more favorable macroscopic outcomes. The long-term risk associated with the synthetic material must be weighed carefully against the comparable short-term efficacy. These results should be interpreted with caution, as preclinical data in rabbit models do not always directly translate to human surgical outcomes.

## Figures and Tables

**Table 1:** The body weight (kg) and the luminal diameter of the duodenum (cm) of rabbits in both test groups before and after surgery.

Rabbits	Before Surgery	2nd Week	4th Week	*p*
Jejunal serosal patch (group I)	The body weight (kg)			
	2.71 ± 0.11	2.91 ± 0.15 *	-	*p* ≤ 0.05
	2.67 ± 0.13	-	3.22 ± 0.12 ***	*p* ≤ 0.001
	The luminal diameter of the duodenum (cm)			
	1 ± 0.1	0.95 ± 0.1 ^†^	-	n.s.
	1.02 ± 0.08	-	0.98 ± 0.13 ^††^	n.s.
ePTFE patch (group II)	The body weight (kg)			
	2.71 ± 0.1	2.94 ± 0.2 *	-	*p* ≤ 0.05
	2.7 ± 0.12	-	3.25 ± 0.13 ***	*p* ≤ 0.001
	The luminal diameter of the duodenum (cm)			
	0.98 ± 0.04	0.9 ± 0.1 ^†††^	-	n.s.
	1 ± 0.07	-	0.96 ± 0.11 ^††††^	n.s.

Note: Data are expressed as mean ± SD for *n* = 5 rabbits per group at each sacrifice interval. Each study group initially consisted of *n* = 10 rabbits, which were equally divided into two sacrifice time points (5 rabbits at the 2nd week and 5 rabbits at the 4th week), ensuring no animal loss during the experiment. * *p* ≤ 0.05, *** *p* ≤ 0.001 compared to values ‘Before surgery’ (Student’s *t*-test); ^†^ *p* = 0.45, ^††^ *p* = 0.58, ^†††^ *p* = 0.14, ^††††^ *p* = 0.52 compared to values ‘Before surgery’ (Student’s *t*-test); n.s. = not statistically significant (*p* > 0.05).

**Table 2:** Wound healing histological scores of animals.

Week (Rabbits)	Jejunal Serosal Patch (Group I)	ePTFE Patch (Group II)	*p*
2nd week (*n* = 5)	11	9	<0.05
4th week (*n* = 5)	19	14	<0.05

Note: The data are presented as the median total histological score (sum of six parameters: epithelialization, collagenization, inflammation, neovascularization, necrosis, and granulation tissue). The maximum possible score per animal is 24. *p* < 0.05 (Mann–Whitney U test). *Wound healing histological scoring system: Epithelialisation (Score 0—Non, 1—Non, 2—Partial, 3—Complete, immature, 4—Complete, mature); Collagenisation (Score 0—Non, 1—Non, 2—Partial, 3—Complete, irregular, 4—Complete, regular); Inflammation (Score 0—Severe, 1—Moderate, 2—Mild, 3—Non, 4—Non); Neovascularisation (Score 0—Non, 1—Non, 2—High-power field (HPF) < 5, 3—HPF 6–10, 4—HPF > 10); Necrosis (Score 0—Extensive, 1—Focal, 2—Non, 3—Non, 4—Non); Granulation tissue (Score 0—Non, 1—Immature, 2—Mild mature, 3—Mod mature, 4—Fully mature)*.

## Data Availability

The original contributions presented in this study are included in the article. Further inquiries can be directed to the corresponding author.

## Abbreviations

The following abbreviations are used in this manuscript:

ePTFE	Expanded polytetrafluoroethylene
E	Epithelium
GC	Foreign-body giant cells
JSP	Jejunal serosal patch
ERCP	Endoscopic retrograde cholangiopancreatography
NSAID	Non-steroidal anti-inflammatory drug
SD	Standard deviation
D	Duodenum
J	Jejunum
P	Patch of expanded polytetrafluoroethylene with graft tissue
M	Mucosa (or re-epithelialized mucosa)
Ms	Muscular
n.s.	Not statistically significant
S	Serosal
ST	Suture tracks
